# Supplementary material for: Mechanistic insights into CO2 conversion chemistry of copper bis-(terpyridine) molecular electrocatalyst using accessible operando spectrochemistry
Source: Nat Commun. 2022 Oct 13;13:6029. doi: 10.1038/s41467-022-33689-9 (PMC9561705; doi:10.1038/s41467-022-33689-9)
Supplement: Supplementary file 1 — Supplementary Information [file 41467_2022_33689_MOESM1_ESM.pdf]

Supplementary Information for:

Mechanistic insights into CO_2 conversion chemistry of copper bis-(terpyridine) molecular electrocatalyst using accessible operando spectrochemistry

Huihui Zhang[#1], Chang Xu[#1,2], Xiaowen Zhan[1,*], Yu Yu[1], Kaifu Zhang[1], Qiquan Luo[1,*], Shan Gao[1,*], Jinlong Yang[2], and Yi Xie[2,*]

[1]School of Chemistry and Chemical Engineering, School of Materials Science and Engineering, Institute of Physical Science and Information Technology, Anhui Province Key Laboratory of Chemistry for Inorganic/Organic Hybrid Functionalized Materials, Key Laboratory of Structure and Functional Regulation of Hybrid Materials of Ministry of Education, Anhui University, 230601 Hefei, Anhui, P.R. China
[2]Hefei National Laboratory for Physical Sciences at Microscale, University of Science and Technology of China, 230026 Hefei, Anhui, P.R. China
[#]These authors contributed equally.
*Correspondence and requests for materials should be addressed to X.W.Z. (xiaowen.zhan@ahu.edu.cn), Q.Q.L. (qluo@ustc.edu.cn), S.G. (shangao@ahu.edu.cn), Y.X. (yxie@ustc.edu.cn).

Table of Contents

1. **Supplementary Fig. S1-S23** 3
2. **Supplementary Table S1-S5** 15
3. **Supplementary Note 1-3** 20
4. **Supplementary References** 32

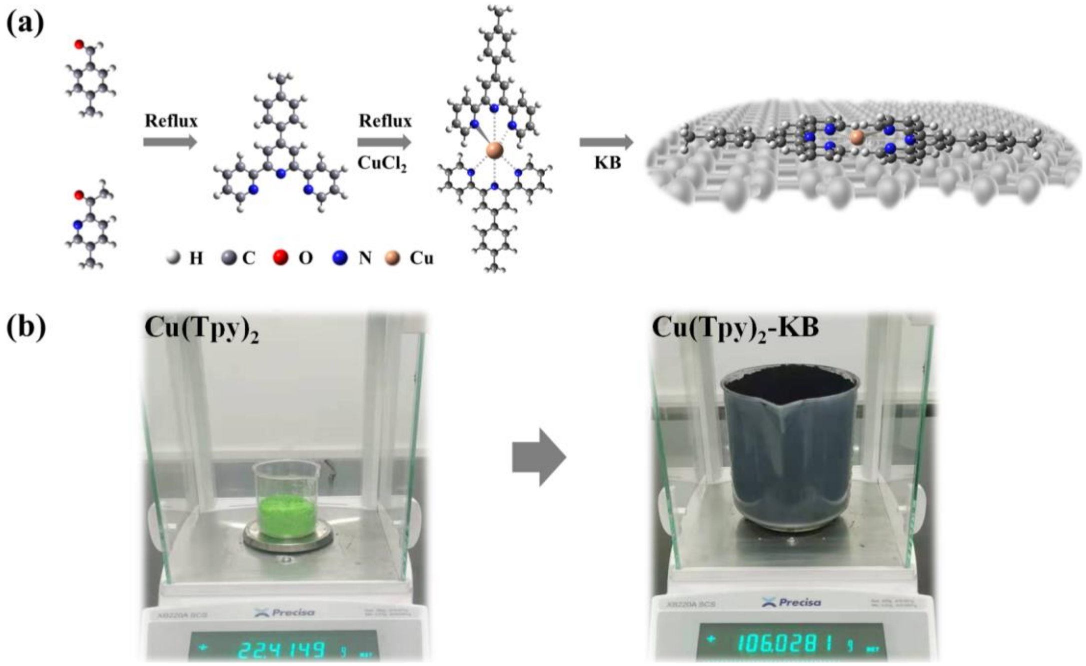

Fig. S1 (a) Schematic illustration for the scalable synthesis and immobilization process of $[Cu(Tpy)_2]Cl_2 \cdot xH_2O$ molecules. **(b)** Photos demonstrating the scale of $[Cu(Tpy)_2]Cl_2 \cdot xH_2O$ and $Cu(Tpy)_2$-KB products synthesized through the approach shown in panel a.

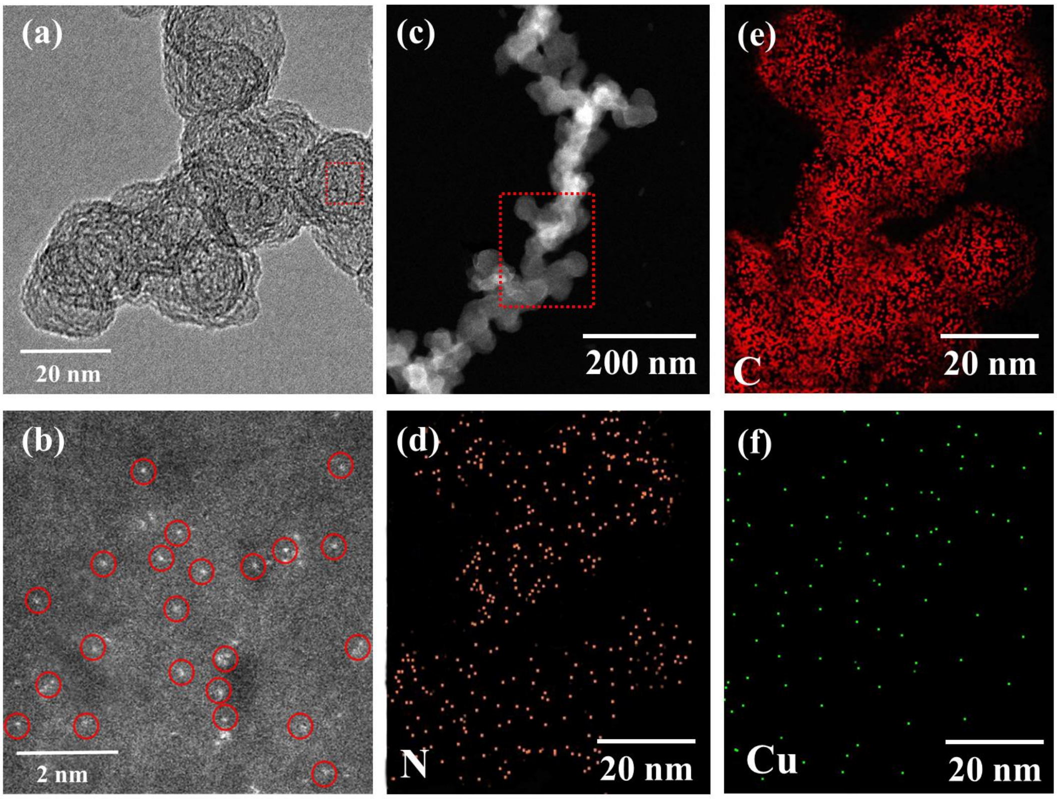

Fig.S2 HAADF-STEM images and corresponding EDX maps of $Cu(Tpy)_2$-KB. The maps displayed are respectively C (red), N (orange), Cu (green).

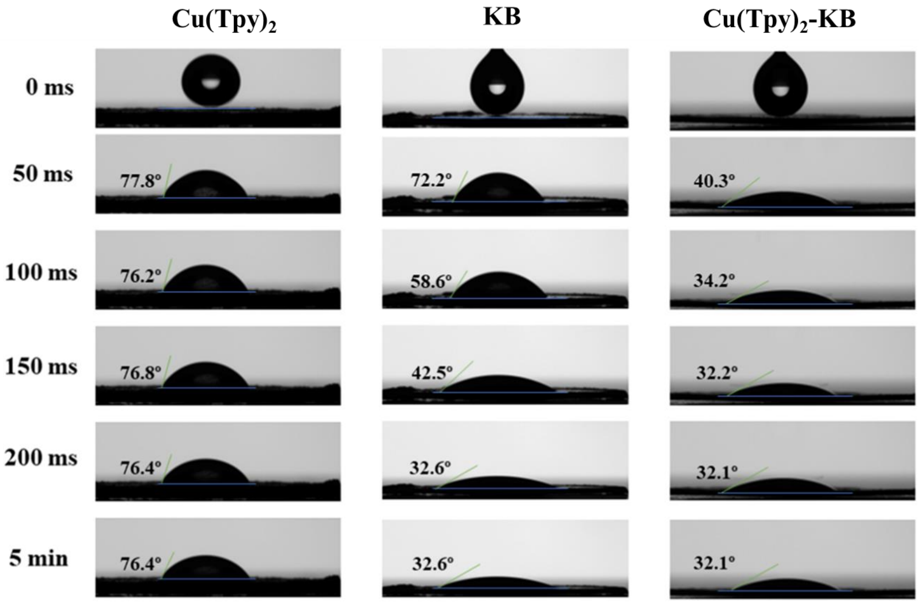

Fig. S3 Contact angles measured for $Cu(Tpy)_2$, KB and Cu(Tpy)-KB electrodes with water drops on top.

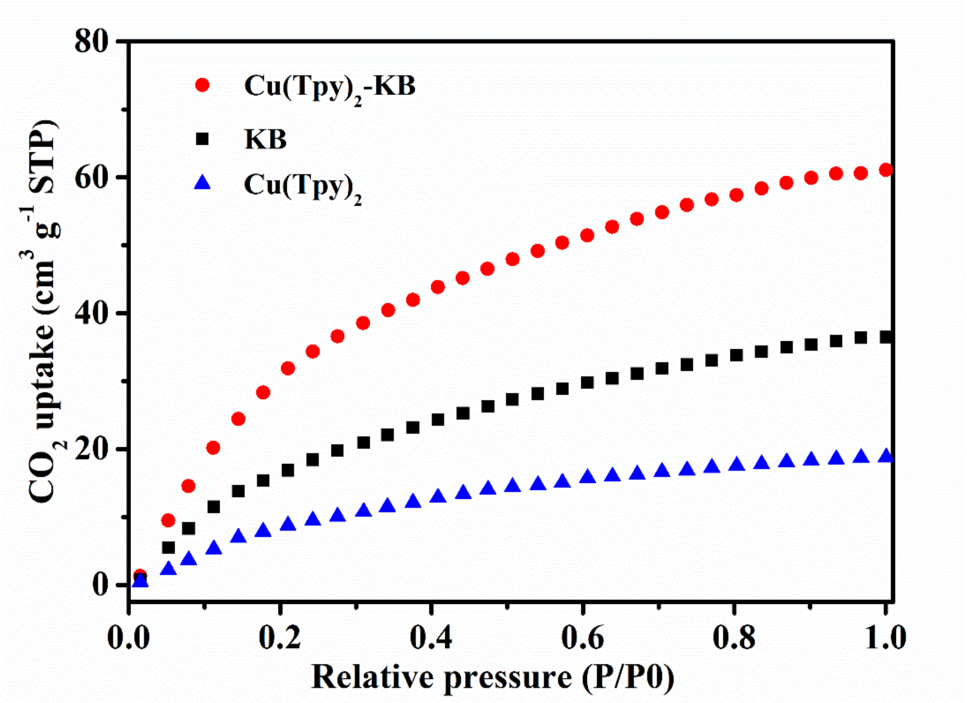

Fig. S4 CO_2 adsorption isotherms of neat $Cu(Tpy)_2$, KB, and $Cu(Tpy)_2$-KB at 25 °C.

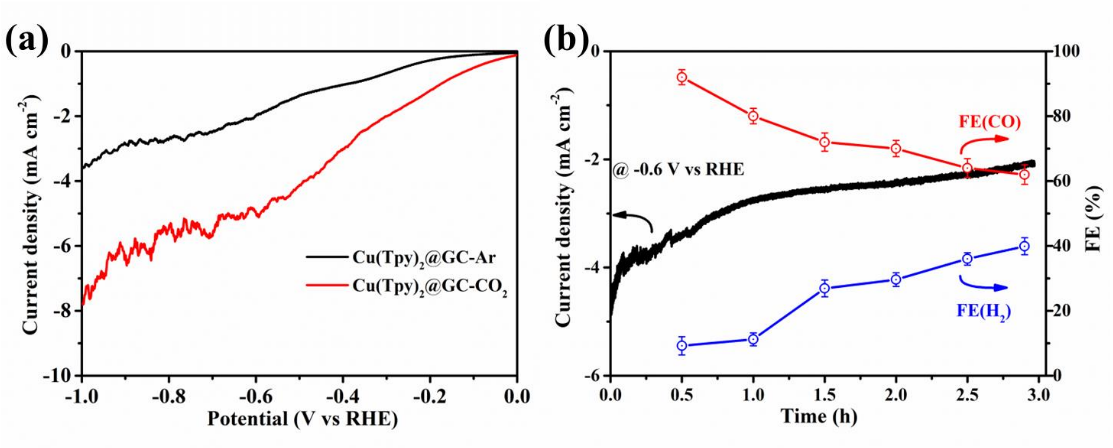

Fig. S5 (a) LSV of $Cu(Tpy)_2$@GC under Ar (black) and CO_2 (red) atmospheres. (b) FEs of $Cu(Tpy)_2$@GC at different applied potentials. The solvent system was DMF/H_2O (95:5, v:v), with 0.1 M of TBAP as supporting electrolyte. Error bars represent the standard deviation from three independent measurements.

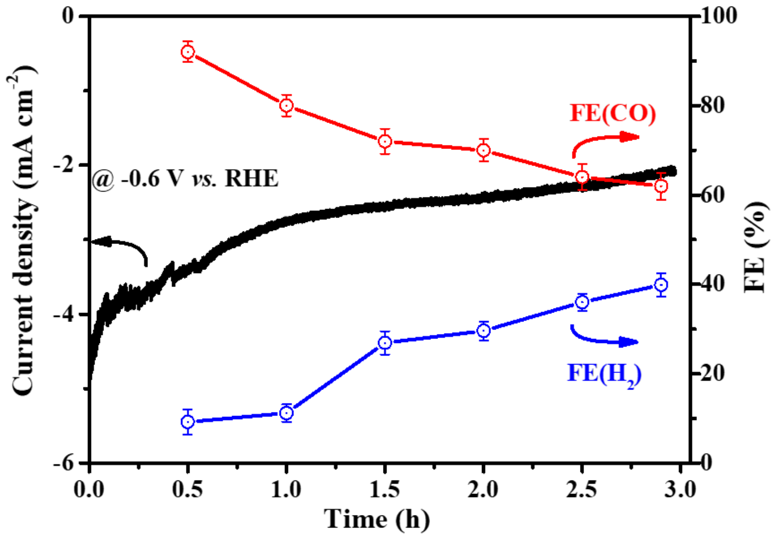

Fig. S6 Chronoamperometry and FEs for CO and H_2 formation at a fixed potential of -0.6 V *vs.* RHE for Cu(Tpy)$_2$@GC under CO_2 atmospheres in DMF/H_2O (95:5, v:v), with 0.1 M of TBAP. Error bars represent the standard deviation from three independent measurements.

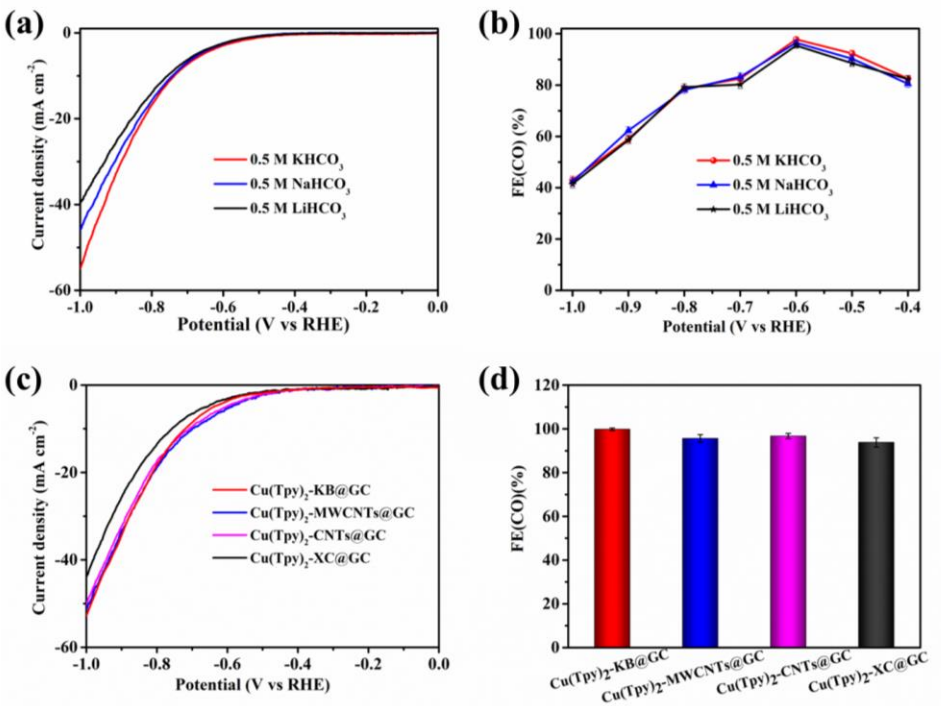

Fig. S7 (a) LSV and (b) FEs(CO) of Cu(Tpy)$_2$-KB@GC acquired using various alkali bicarbonate CO_2-saturated aqueous solutions. (c) LSV and (d) FE(CO)s at -0.6 V *vs.* RHE of Cu(Tpy)$_2$ catalysts immobilized on different carbon supports including Ketjen black (KB), multi wall carbon nanotubes (MWCNTs), single-wall carbon nanotubes (CNTs), and Vulcan carbon-72R (XC) using a CO_2-saturated 0.5 M $KHCO_3$ electrolyte. Error bars represent the standard deviation from three independent measurements.

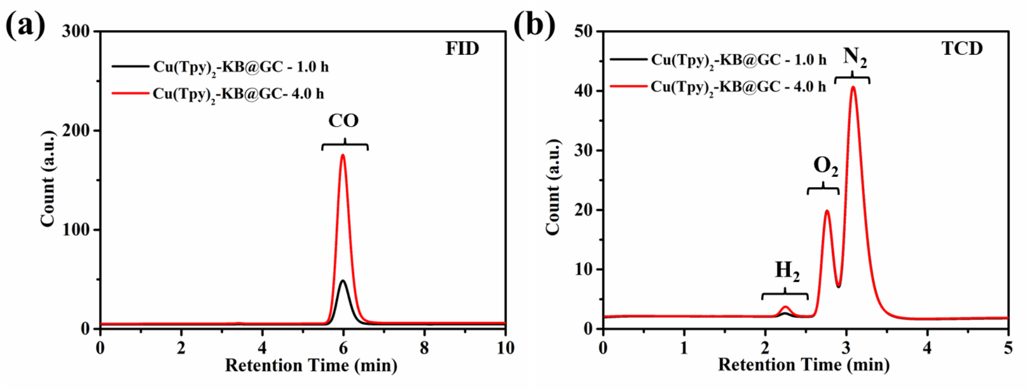

Fig. S8 Gas chromatograms profiles of CO and H_2. (a) and (b) are respectively FID and TCD signals captured on $Cu(Tpy)_2$-KB@GC electrode at an overpotential of -0.40 V *vs*. RHE in a 0.5 M CO_2-saturated $KHCO_3$ aqueous solution.

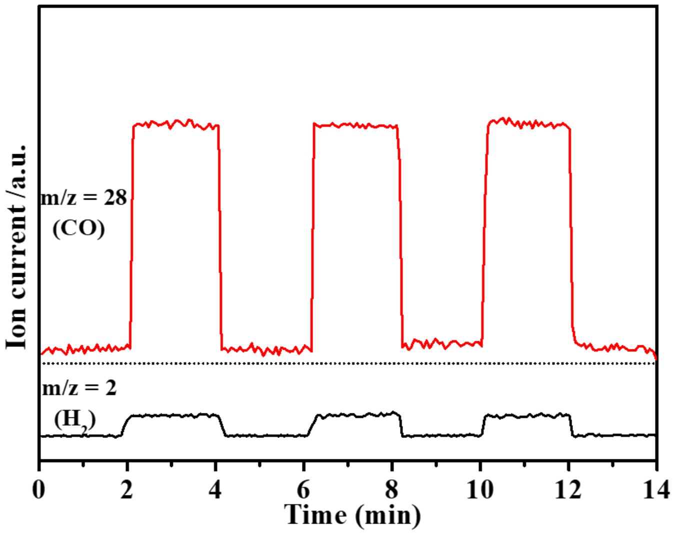

Fig. S9 Online differential electrochemical mass spectrometry (DEMS) measurements profile for the CO_2RR of $Cu(Tpy)_2$-KB@GC electrode. The DEMS measurements were conducted during the controlled-potential electrolysis at -0.4 V *vs*. RHE for three times. The two strongest signals of *m*/*z*=28 and of *m*/*z*=2 was detected and were assigned to CO and H_2 products, respectively.

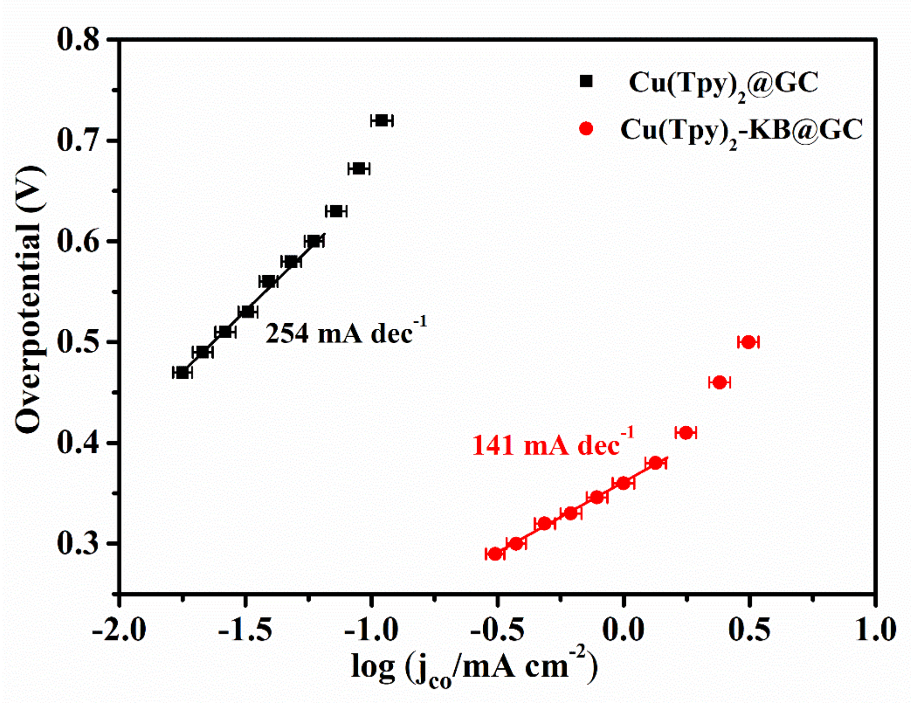

Fig. S10 Tafel plots of $Cu(Tpy)_2$@GC and $Cu(Tpy)_2$-KB@GC. Error bars represent the standard deviation from three independent measurements.

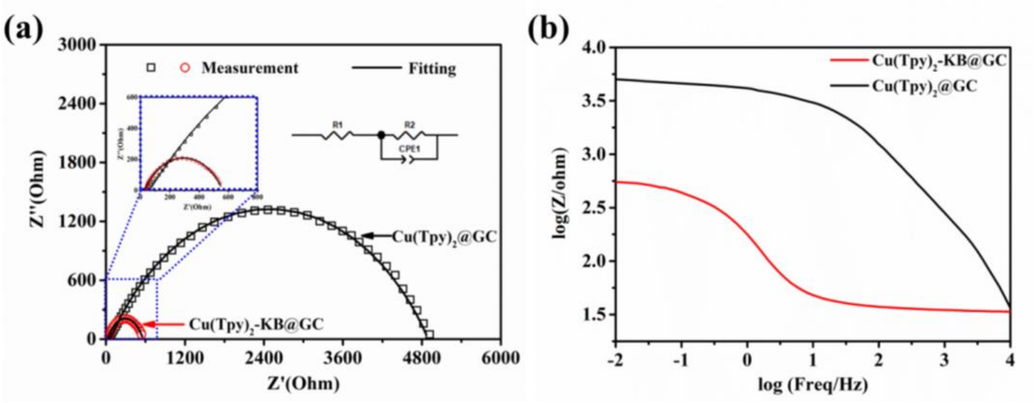

Fig. S11 Nyquist plots of EIS (a) and Bode plot (b) of $Cu(Tpy)_2$@GC and $Cu(Tpy)_2$-KB@GC in CO_2-saturated 0.5 M $KHCO_3$ electrolyte; the inset is the electrical equivalent circuit used to simulated the experimental impedance data.

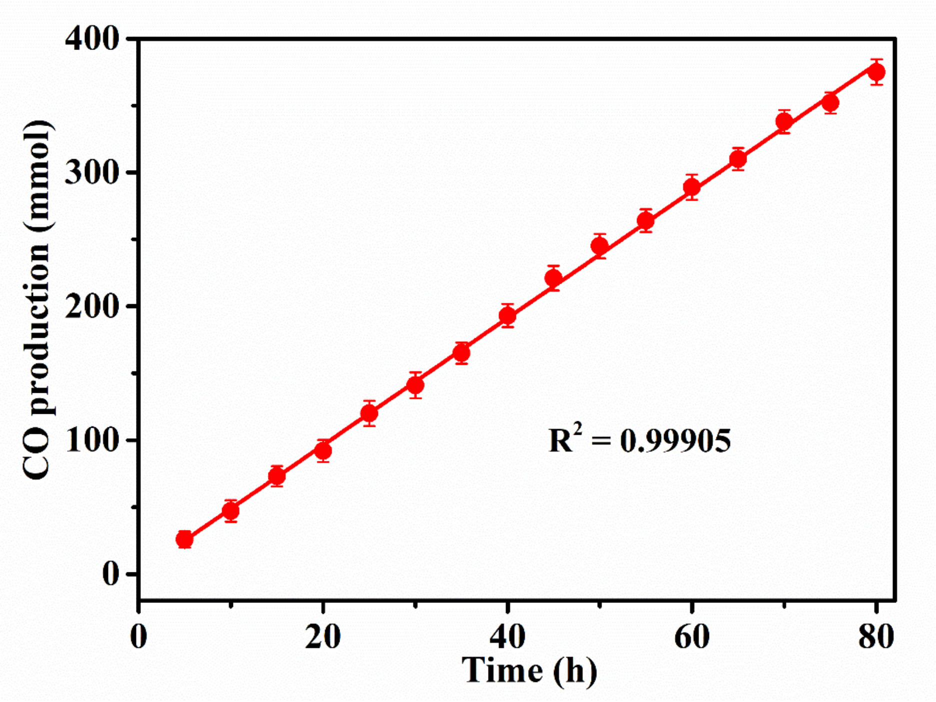

Fig. S12 CO production at a fixed potential of -0.6 V *vs.* RHE for Cu(Tpy)2-KB@GC. The error bars represent standard deviation of three measurements

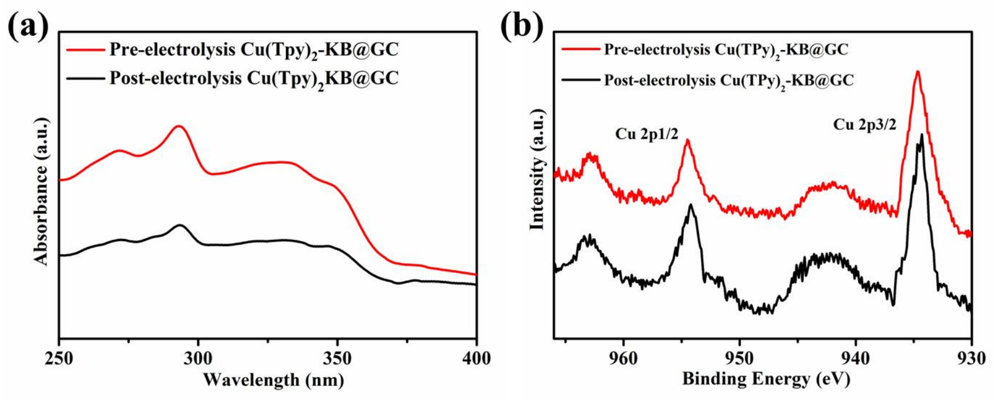

Fig. S13 (a) FT-IR spectra and (b) Cu2*p* XPS spectra of Cu(Tpy)2-KB@GC before and after electrolysis.

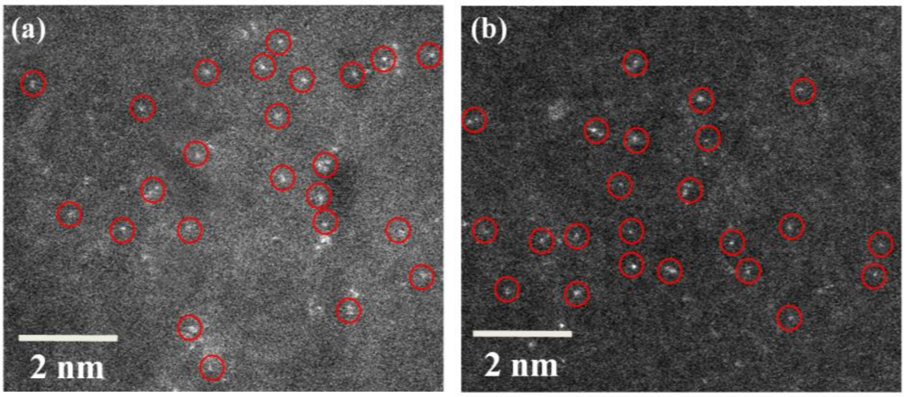

Fig. S14 HAADF-STEM images of Cu(Tpy)$_2$-KB@GC before (a) and after (b) electrolysis at -0.6 V *vs.* RHE

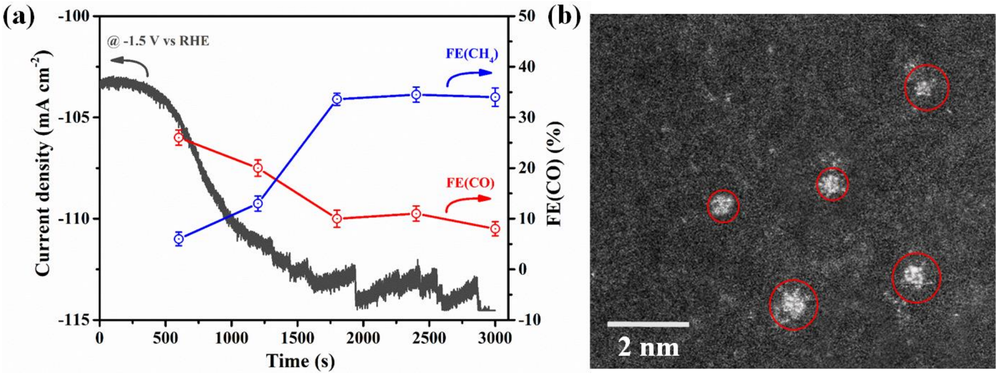

Fig. S15 (a) Chronoamperometry and FEs for CO and CH_4 formation at a fixed potential of -1.5 V *vs.* RHE for Cu(Tpy)$_2$-KB@GC. (b) HAADF-STEM images of Cu(Tpy)$_2$-KB@GC after electrolysis at -1.5 V *vs.* RHE. Error bars represent the standard deviation from three independent measurements.

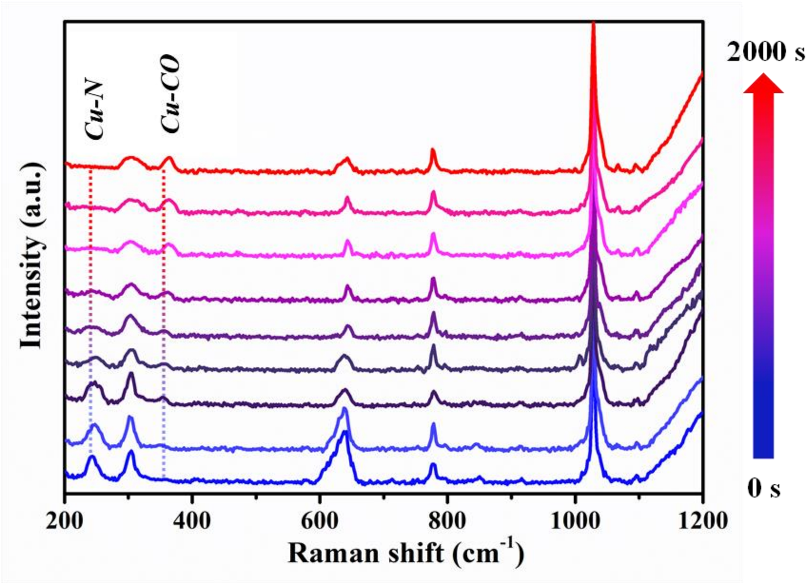

Fig. S16 Time-dependent operando Raman spectra of Cu(Tpy)$_2$-KB@GC using a CO_2-saturated 0.5 M $KHCO_3$ solution at a fixed potential of -1.5 V *vs.* RHE.

We collected operando Raman spectra of Cu(Tpy)$_2$-KB@GC under the same potential. As shown in **Fig. S16**, the peaks at 247 and 354 cm^{-1} could be assigned to the Cu-N bonding environment in the hybrid and *CO, respectively. The other peaks could be assigned to the C=C and C-H bonding environment in the Cu(Tpy)$_2$. One important takeaway from **Figure S16** was that the intensity of Cu-N peak gradually decreased before disappearance. This could serve as an evidence that the electrochemical CO_2RR led to the breaking of Cu-N bonds under high potentials (i.e., -1.5 V *vs.* RHE), with the copper sites converting into agglomerates of metallic copper clusters. This was also consistent with the shift of the *CO peak to higher wavenumbers starting from 1250 s, signalizing the *CO adsorption on Cu sites of metallic Cu clusters rather than on the original coordinated Cu^{2+} sites. Therefore, when the potential was too high, i.e., -1.5 V *vs.* RHE, major conversion of Cu^{2+} sites to metallic Cu nanoclusters was evident.

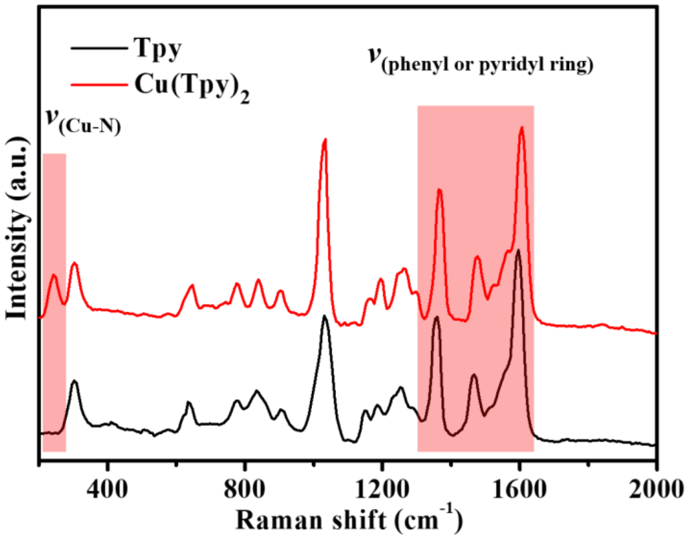

Fig. S17 Raman spectra of solid Tpy and Cu(Tpy)$_2$ in the wavenumber range 2000-200 cm^{-1}. From the vibrational analysis of both Tpy and Cu(Tpy)$_2$, the Raman line at ~247 cm^{-1} is instead assigned to ν(Cu-N) in Cu(Tpy)$_2$. The vibration mode of phenyl or pyridyl ring at 1300-1700 cm^{-1} are shifted to the right for Cu(Tpy)$_2$.

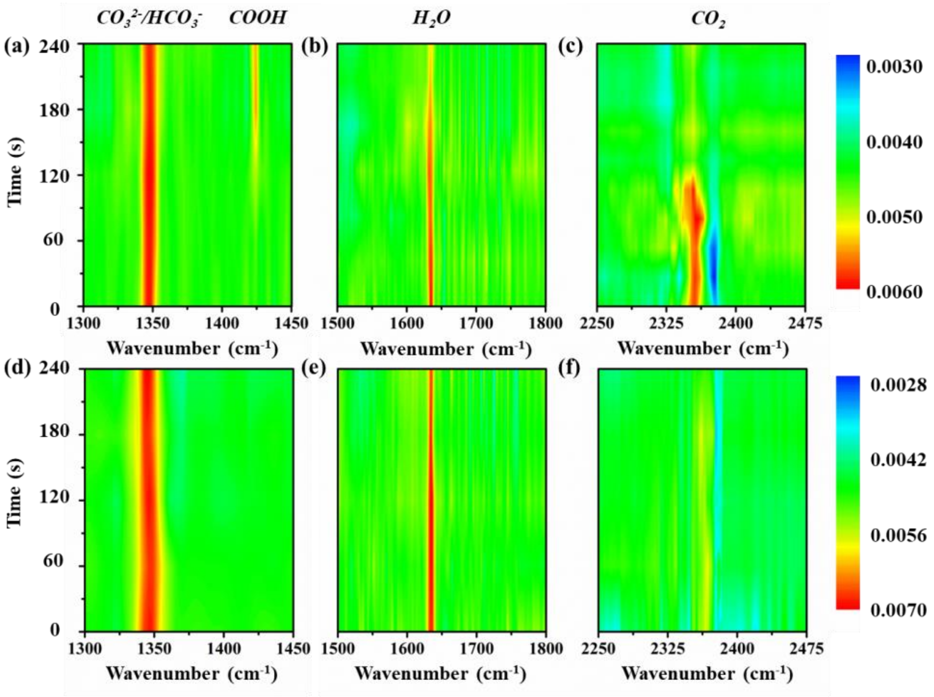

Fig. S18 Time-dependent *operando* FT-IR spectra of Cu(Tpy)$_2$-KB@GC under electrocatalytic conditions. The spectra were measured using (a-c) a CO_2-saturated 0.5 M $KHCO_3$ solution and (d-f) a Ar-saturated 0.5 M $KHCO_3$ solution, respectively. The potential was controlled at -0.45 V *vs.* RHE. The results are displayed in three scanning subsections, namely (a, d) 1300-1450 cm^{-1}, (b, e) 1500-1800 cm^{-1}, (c, f) 2250-2475 cm^{-1}.

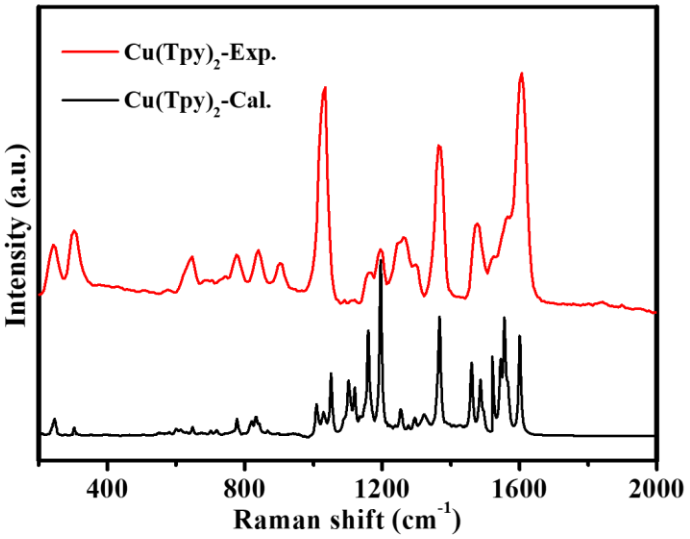

Fig. S19 Raman spectra of Cu(Tpy)$_2$. Experimental (top, excited at 632.8 nm) and calculated (bottom, ×0.96, B3LYP-D3/6-31G**)

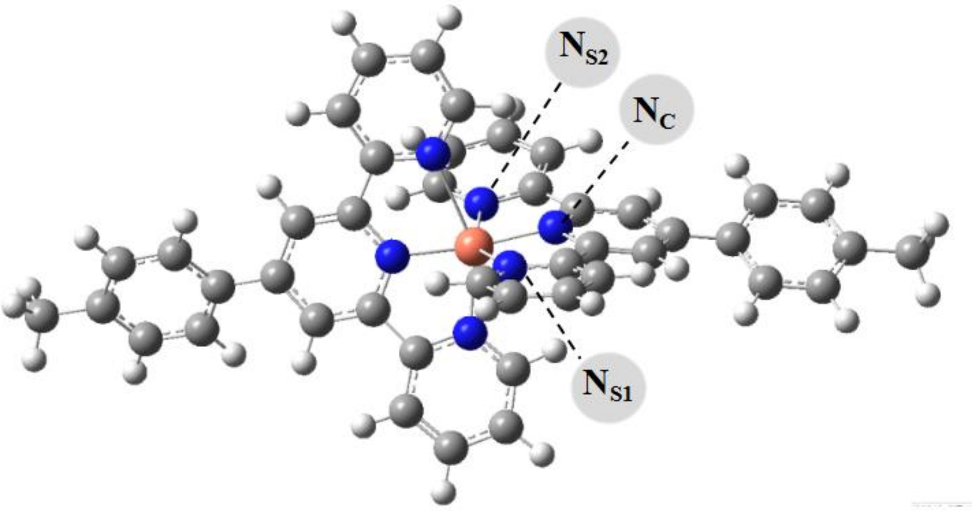

Fig. S20 Mulliken charges of *COOH on different active sites (central and two side nitrogen atoms) and their respective Gibbs free energies (ΔG) calculated for the reaction, $CO_2 + H^+ + e^- + * \rightarrow *COOH$.

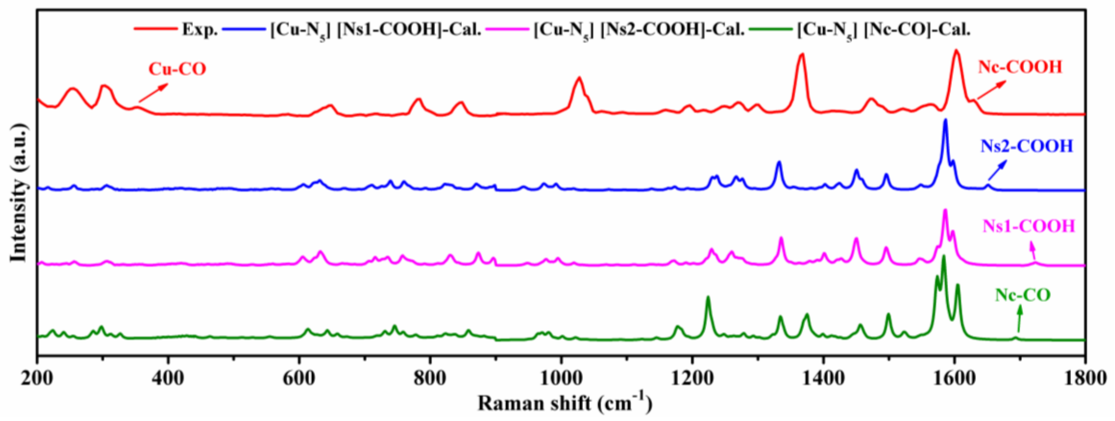

Fig. S21 Comparisons among the DFT-calculated and *in-situ* measured Raman spectra of $Cu(Tpy)_2$. The experimental line was extracted from the top spectra in panels a-d of Fig. 3 at the end of 2.5 s.

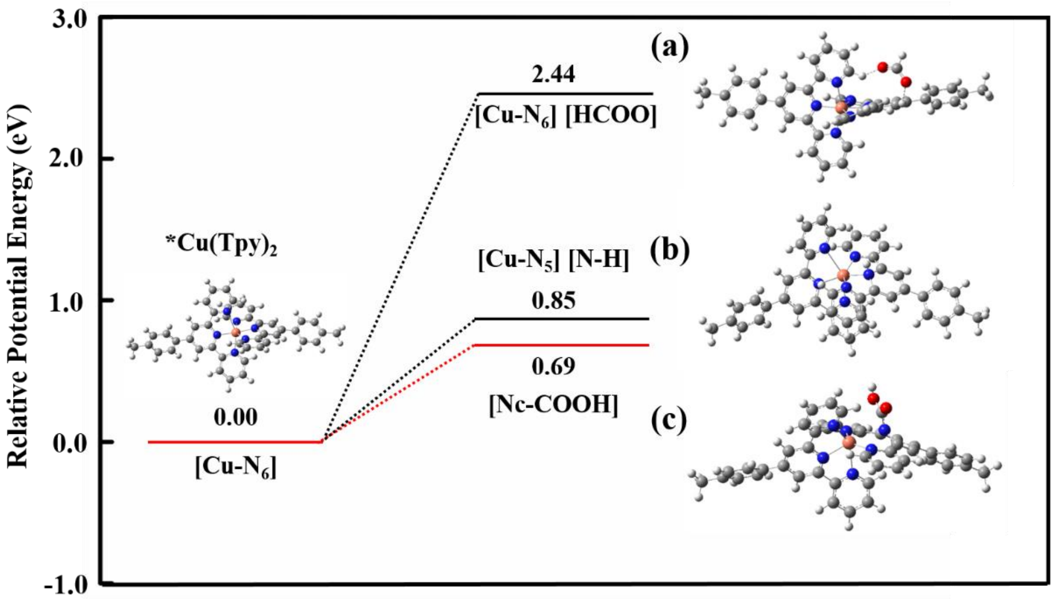

Fig. S22 Calculated Gibbs free energies for different adsorption pathways, namely (a) CO_2 (g) + H^+ + e^- + * → HCOO(*). (b) H^+ + e^- + * → H(*), and (c) CO_2 (g) + 2 (H^+ + e^-) + * → COOH(*).

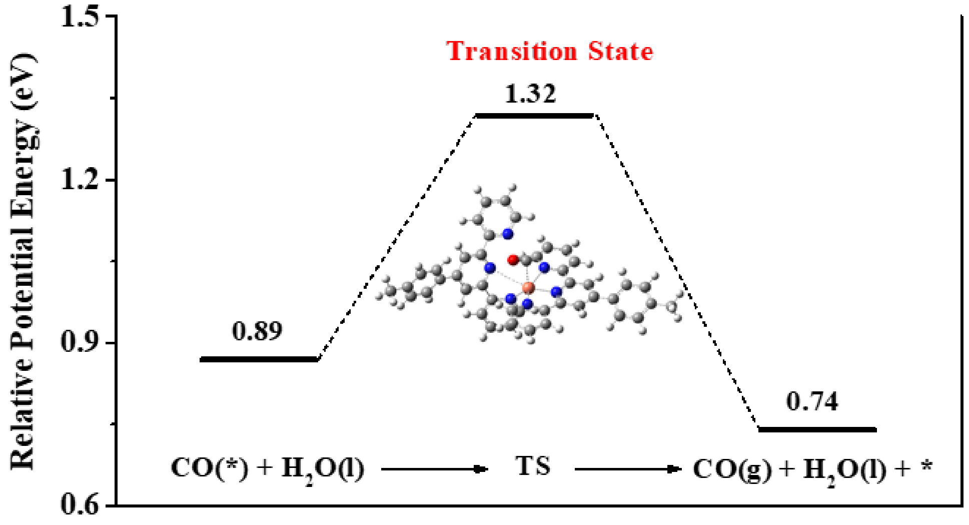

Fig. S23 Reaction path for the electrocatalytic CO_2RR to CO on Cu(Tpy)$_2$-KB@GC catalyst with a transition state considered for the CO desorption step.

The first protonation step is the potential limiting step in this reaction and forms the adsorbed *COOH species. And the formed massive *COOH stay at catalysts and

are ready for the next step reaction. Our Raman spectrum captured this species. Combining with the DFT calculation, this adsorbed configuration was identified as [Nc-COOH]. Following the second electron-proton step, *CO was formed. Inspired by the reviewer's insightful suggestion, we have investigated this process in detail and found that an energy barrier of 0.48 eV was required and was facile to conquer under the working condition. The reaction path containing this transition state was given in Fig. S19. The barriered CO desorption allowed the adsorbed CO* to be observed experimentally. This was mainly because the dynamic process occurred in this step consists of both Cu-CO bond breakage and Cu-N bond formation. Additionally, there were many identical active sites available in Cu(Tpy)$_2$-KB@GC, all of which would contribute to the signals of these key intermediates, thereby enabling the successful observation of intermediates via a widely-accessible regular Raman spectroscopy.

Supplementary Table S1 Electrocatalytic performance comparison of the Cu(Tpy)$_2$-KB@GC hybrid with representative noble-metal-free catalysts for CO_2RR-to-CO in H-cells with 0.5 M aq. $KHCO_3$ electrolyte.

Catalysts	TOF(CO) s^{-1}	FE(CO)	Stability @ V *vs.* RHE	Ref.
Cu(Tpy)$_2$-KB@GC	9.4	99.5%	80 h @ -0.60	This study
NiPc–OMe MDE	12	99%	40 h @ -0.61	1
CoPc-CN/CNT	4.1	98%	10 h @ -0.63	2
Co-TTCOF	1.28	91.3%	40 h @ -0.70	3
CoCp2@MOF-545-Co	0.22	82%	8 h @ -0.70	4
CoPPc/CNT	1.36	~80%	24 h @ -0.54	5
Co-TPP/CNT	4.77	~80%	11 h @ -0.55	6
S$_2$Co-G	2.75	~92%	/	7
CoPc2	6.8	~93%	11 h @ -0.68	8
CoPc-P4VP	4.8	~90%	6 h @ -0.73	9
CCG/CoPc -A	5	~80%	30 h @ -0.69	10

Supplementary Table S2 Experimental (top, excited at 632.8 nm), calculated (bottom, ×0.96, B3LYP-D3/6-31G**) Raman bands (in cm^{-1}) and assignments of $Cu(Tpy)_2$.

Experimental	Calculated	Assignment	
v	*v* × 0.96	Vibrational motion description	
244	245	*v* (Cu-N)	Metal-ligand stretching
299	304	Γ(ring-ring)	Ring-ring wagging (tpy)
649	648	Δ(ring)	Phenyl + pyridyl
781	778	Δ(ring)	Phenyl + pyridyl
842	841	Δ(ring)	Phenyl + pyridyl
902	899	Δ(ring)	Phenyl + pyridyl
1029	1007,1031,1051	δ(CH) + *v*(ring)	Phenyl + pyridyl
1162	1160	δ(CH) + *v*(ring)	Phenyl + pyridyl
1197	1194	δ(CH) + *v*(ring)	Phenyl + pyridyl
1259	1256	δ(CH) + *v*(ring)	Phenyl + pyridyl
1303	1300	δ(CH) + *v*(ring)	Phenyl + pyridyl
1366	1365	*v*(ring)	C-C and/or C-N stretching
1480	1461,1484	*v*(ring)	C-C and/or C-N stretching
1524	1518,1541	*v*(ring)	C-C and/or C-N stretching
1568	1562	*v*(ring)	C-C and/or C-N stretching
1607	1602	*v*(ring)	C-C and/or C-N stretching

Symbol *v* denotes stretch, *δ* in-plane and out-of-plane bending and , Γ, Δ for in-plane and out-of-plane deformation of phenyl or pyridyl ring.

Supplementary Table S3 Experimental (top, excited at 632.8 nm), calculated (bottom, ×0.96, B3LYP-D3/6-31G**) Raman bands (in cm^{-1}) and assignments of $Cu(Tpy)_2$-COOH .

Experimental	Calculated	Assignment	
v	*v* × 0.96	Vibrational motion description	
254	239	*v* (Cu-N)	Metal-ligand stretching
300	302	Γ(ring-ring)	Ring-ring wagging (tpy)
580	582	Δ(ring)	Phenyl + pyridyl
648	645,649	Δ(ring)	Phenyl + pyridyl
694	700	Δ(ring)	Phenyl + pyridyl
782	781,806	Δ(ring)	Phenyl + pyridyl
848	843	Δ(ring)	Phenyl + pyridyl
1025	1000,1031,1049	δ(CH) + *v*(ring)	Phenyl + pyridyl
1067	1070	δ(CH) + *v*(ring)	Phenyl + pyridyl
1093	1095	δ(CH) + *v*(ring)	Phenyl + pyridyl
1196	1204	δ(CH) + *v*(ring)	Phenyl + pyridyl
1271	1238,1263,1294	*v*(ring)	C-C and/or C-N stretching
1370	1380	*v*(ring)	C-C and/or C-N stretching
1472	1470,1483	*v*(ring)	C-C and/or C-N stretching
1519	1520	*v*(ring)	C-C and/or C-N stretching
1563	1552	*v*(ring)	C-C and/or C-N stretching
1602	1606	*v*(ring)	C-C and/or C-N stretching
1628	1631	*v*(N_c-COOH)	C-O stretching

Symbol *v* denotes stretch, *δ* in-plane and out-of-plane bending and , Γ, Δ for in-plane and out-of-plane deformation of phenyl or pyridyl ring.

Supplementary Table S4 Experimental (top, excited at 632.8 nm), calculated (bottom, ×0.96, B3LYP-D3/6-31G**) Raman bands (in cm^{-1}) and assignments of $Cu(Tpy)_2$-CO .

Experimental	Calculated	Assignment	
v	*v* × 0.96	Vibrational motion description	
253	251	*v* (Cu-N)	Metal-ligand stretching
303	310	Γ(ring-ring)	Ring-ring wagging (tpy)
354	356	*v* (Cu-CO)	Metal-C=O stretching
580	572,592	Δ(ring)	Phenyl + pyridyl
648	644	Δ(ring)	Phenyl + pyridyl
783	752,769,809	Δ(ring)	Phenyl + pyridyl
848	837,863	Δ(ring)	Phenyl + pyridyl
1029	1019,1028	δ(CH) + *v*(ring)	Phenyl + pyridyl
1158	1155	δ(CH) + *v*(ring)	Phenyl + pyridyl
1195	1185	δ(CH) + *v*(ring)	Phenyl + pyridyl
1217	1220	δ(CH) + *v*(ring)	Phenyl + pyridyl
1246	1236	*v*(ring)	C–C and/or C–N stretching
1273	1273	*v*(ring)	C–C and/or C–N stretching
1299	1284,1297	*v*(ring)	C–C and/or C–N stretching
1368	1369	*v*(ring)	C–C and/or C–N stretching
1420	1422	*v*(ring)	C–C and/or C–N stretching
1471	1471	*v*(ring)	C–C and/or C–N stretching
1524	1530	*v*(ring)	C–C and/or C–N stretching
1563	1560	*v*(ring)	C–C and/or C–N stretching
1604	1606	*v*(ring)	C–C and/or C–N stretching

Symbol *v* denotes stretch, *δ* in-plane and out-of-plane bending and , Γ, Δ for in-plane and out-of-plane deformation of phenyl or pyridyl ring.

Supplementary Table S5 Mulliken charges of *COOH on different active sites (central and two side nitrogen atoms) and their respective Gibbs free energies (ΔG) calculated for the reaction, $CO_2 + H^+ + e^- + * \rightarrow *COOH$.

Different sites	Mulliken Charges/e	ΔG/eV
Nc	-0.73	0.69
N_{S1}	-0.50	0.97
N_{S2}	-0.60	0.73
Cu	+0.86	-

Supplementary Note 1

^{1}H NMR (600 MHz, DMSO-d_6), δ (ppm): 8.78-8.76 (J = 8.77, d, 2H), 8.70 (s, 2H), 8.68-8.66 (J = 8.67, d, 2H), 8.16-8.04 (J = 8.04, d, 2H), 7.84-7.82 (J = 7.83, d, 2H), 7.55-7.53 (J = 7.53, d, 2H), 7.42-7.40 (J = 7.53, d, 2H), 2.41 (s, 3H). ^{13}C NMR (150 MHz, DMSO-d_6), δ (ppm): 156.17, 155.52, 139.76, 138.00, 135.07, 130.54, 125.05, 121.46, 118.16, 21.35. FT-IR (KBr, cm^{-1}): 3433(w), 3019(w), 2923(w), 1602(s), 1584(vs), 1564(vs), 1465(vs), 1389(vs), 1264(m), 1039(m), 886(m), 820(s), 790(vs), 733(s), 685(m). MS (ESI) m/z: calcd for $[M+H]^+$, 324.1507; Found, 324.1493.

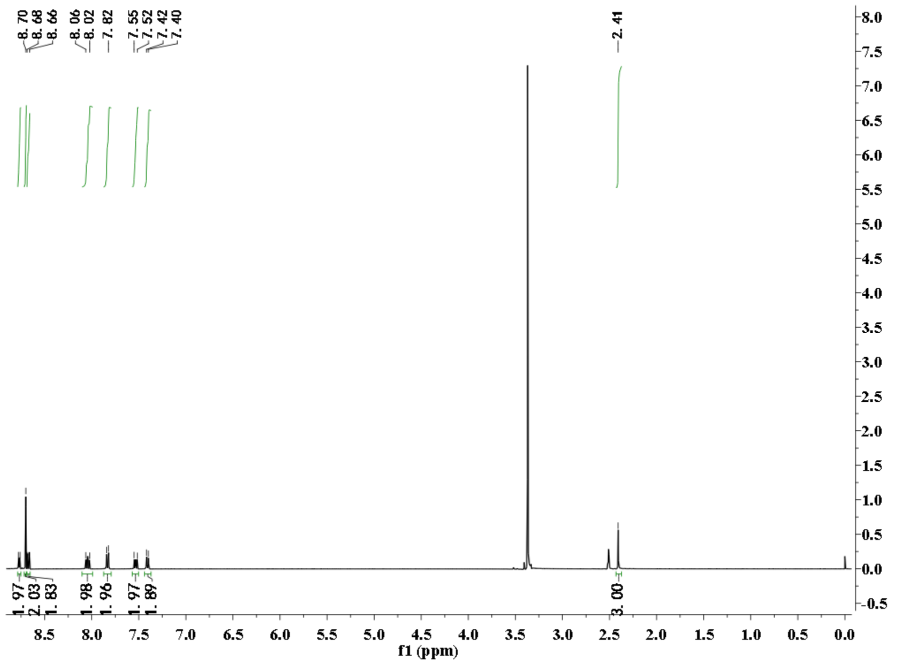

S1 ^{1}H NMR spectrum of Tpy (in DMSO-d_6)

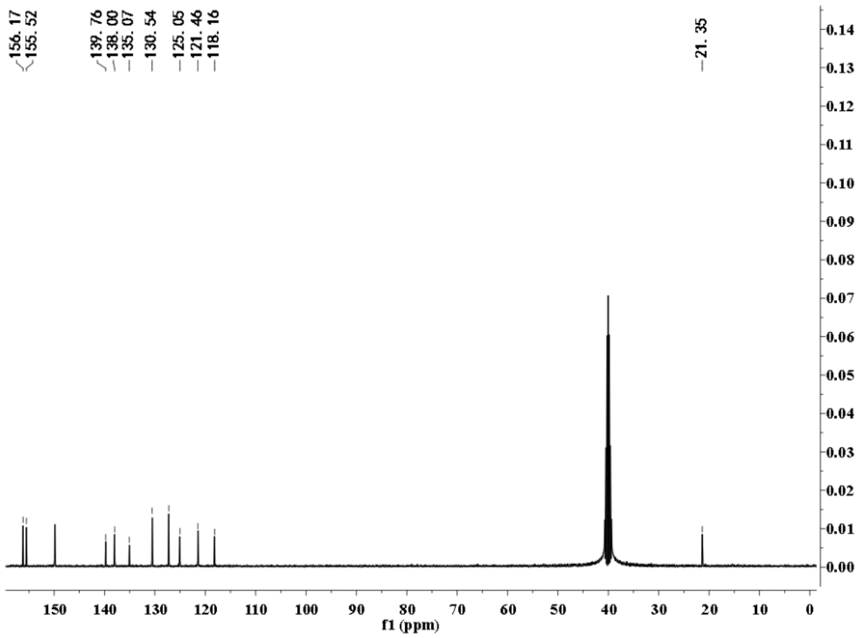

S2 ^{13}C NMR spectrum of Tpy (in DMSO-d_6)

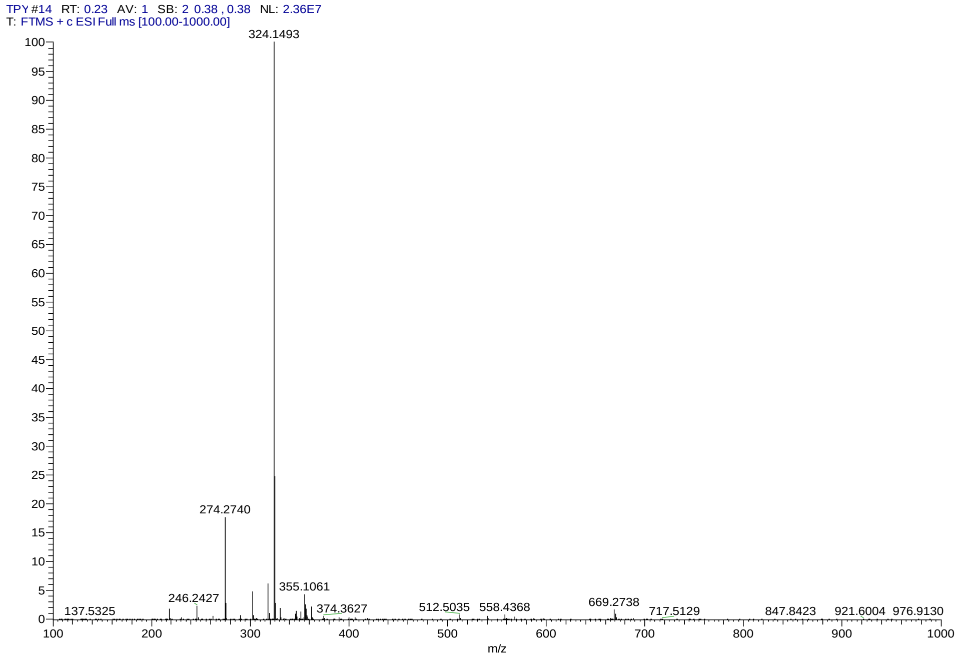

S3 ESI-Mass spectrum of Tpy

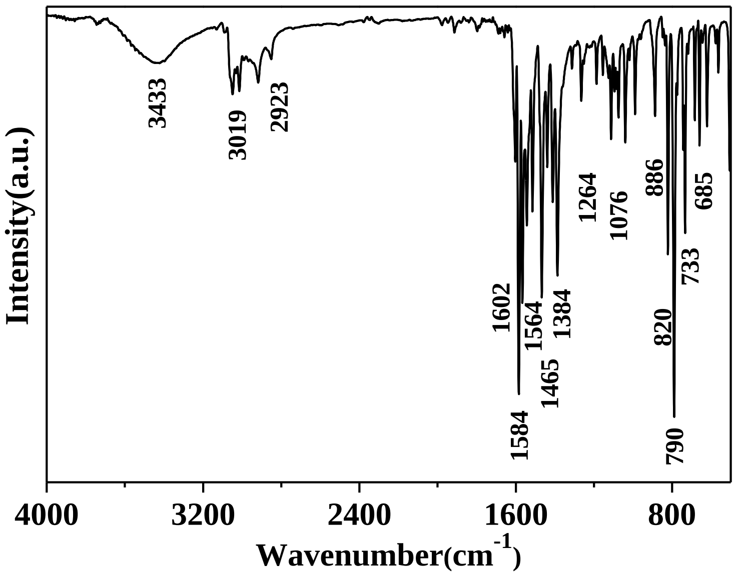

S4 FT-IR spectrum of Tpy in KBr

Supplementary Note 2

Anal. Calcd. for $C_{44}H_{34}Cl_2CuN_6$: C, 67.65; H, 4.39; N, 10.76. Found: C, 67.80; H, 4.34; N, 10.85%. FT-IR (KBr, cm^{-1}). 3425(w), 3063(w), 2916(w), 2849(w), 1603(vs), 1554(s), 1477(vs), 1434(vs), 1252(m), 1159(m), 1021(m), 830(s), 791(vs), 725(s).

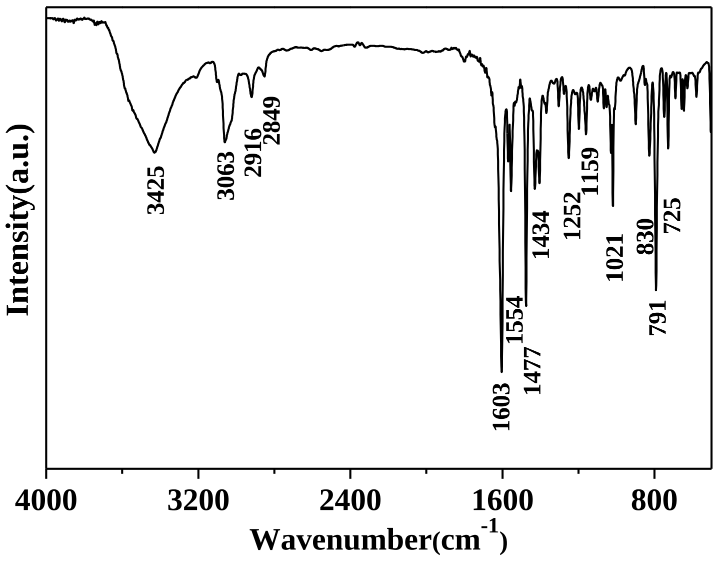

S5 FT-IR of $[Cu(Tpy)_2]Cl_2{\bullet}xH_2O$ in KBr

Supplementary Note 3

Cartesian Coordinates of Calculated Structures:

$Cu(Tpy)_2$

C	-6.14688700	-0.22372200	-0.64674800
C	-6.59806600	-0.67991100	-1.90039500
C	-7.95038200	-0.67184600	-2.22814500
C	-8.91623700	-0.20235700	-1.32835200
C	-8.47165200	0.25407700	-0.07990400
C	-7.12169600	0.24519100	0.25576100
N	-1.95569600	-0.25178700	0.38204300
C	-2.49096900	-1.20018600	-0.46319500
C	-3.84034600	-1.21591200	-0.79639600
C	-4.71898200	-0.24431700	-0.28942900
C	-4.15303400	0.72133100	0.57885100
C	-2.80391700	0.69433900	0.89500500
C	-0.17939500	2.51871800	2.61651300
N	-0.83218700	1.66558600	1.82715800
C	-2.18002900	1.67451800	1.81115800
C	-2.90856100	2.56158000	2.62512700
C	-2.21993000	3.45331400	3.43810100
C	-0.82076300	3.44262700	3.43857200
C	0.74660600	-2.61681200	-1.26396800
C	0.49416700	-3.86735800	-1.80803000
C	-0.84463400	-4.27963400	-1.92501000
C	-1.85456200	-3.43414900	-1.49593800
C	-1.52475700	-2.17141200	-0.96849900
N	-0.22265800	-1.78122800	-0.86499300
C	-10.37835900	-0.15940200	-1.70062900
H	-5.87662200	-1.01886500	-2.63788100
H	-8.26236700	-1.02669400	-3.20769200
H	-9.19830800	0.61039200	0.64669200
H	-6.82026700	0.57203400	1.24657800
H	-4.21867400	-1.99021100	-1.45441600
H	-4.78080700	1.51339700	0.96854700
H	0.90623500	2.45340700	2.57464600
H	-3.99232900	2.54266800	2.63623900
H	-2.76688800	4.14491700	4.07234900
H	-0.24885900	4.12365100	4.05997500
H	1.76091100	-2.25022400	-1.12891500
H	1.31303300	-4.50195100	-2.12814100
H	-1.08670500	-5.25803000	-2.32904000
H	-2.89069800	-3.75125200	-1.53755000

Cu	-0.00009200	0.00610400	0.13853100
C	6.14979300	0.19609000	-0.64128900
C	6.85709500	1.36768300	-0.97662000
C	8.20654200	1.32640000	-1.30933400
C	8.91981700	0.11924300	-1.31756800
C	8.22097400	-1.04815500	-0.98559800
C	6.86887600	-1.01452900	-0.65625700
N	1.95559000	0.27088000	0.37062300
C	2.49391400	1.18408900	-0.51126500
C	3.84619700	1.18815700	-0.83645500
C	4.72120300	0.23656300	-0.28898700
C	4.14994600	-0.69664800	0.61238500
C	2.80103700	-0.65363500	0.92455100
C	0.17088800	-2.37555400	2.74042000
N	0.82753800	-1.56518500	1.91030500
C	2.17508100	-1.58254000	1.89272400
C	2.89993700	-2.43433800	2.74631000
C	2.20737900	-3.28059500	3.60360700
C	0.80843100	-3.26149700	3.60604500
C	-0.74480700	2.54563200	-1.40037800
C	-0.49282400	3.76454800	-2.01195000
C	0.84600800	4.17138200	-2.14903700
C	1.85572900	3.35283000	-1.67098400
C	1.52703900	2.11975100	-1.07602200
N	0.22455200	1.73360300	-0.95540000
C	10.39317300	0.08768700	-1.64365800
H	6.34774400	2.32645000	-0.94904400
H	8.72206300	2.25138300	-1.55751200
H	8.74173500	-2.00283000	-0.99847300
H	6.35342200	-1.94623300	-0.44306500
H	4.21444200	1.89617300	-1.57027600
H	4.78981800	-1.43093300	1.08649600
H	-0.91441300	-2.30652100	2.69576100
H	3.98379300	-2.42342300	2.75372000
H	2.75148200	-3.94364600	4.26998900
H	0.23381300	-3.90783600	4.26109600
H	-1.75897300	2.18602700	-1.24695100
H	-1.31183000	4.37940700	-2.36810100
H	1.08774100	5.12593300	-2.60675000
H	2.89159900	3.66865700	-1.72719000
H	-11.01943900	-0.31250300	-0.82663300
H	-10.62685000	-0.92657600	-2.44046300
H	-10.64998500	0.81134000	-2.13504100
H	10.70255500	-0.89656500	-2.00823400

H	11.00149700	0.31334800	-0.75832200
H	10.65056100	0.82755400	-2.40847600

$Cu(Tpy)_2$-COOH

C	-5.93227300	-1.40304500	0.32037300
C	-7.11299400	-0.67846300	0.55090300
C	-8.35959000	-1.28670400	0.43488300
C	-8.47788100	-2.63821100	0.08499300
C	-7.29965100	-3.35913600	-0.14993100
C	-6.04992800	-2.75722500	-0.03358900
N	-2.06050000	0.43635100	0.62148100
C	-3.12375200	1.12794100	0.14510900
C	-4.39659200	0.56860000	0.04611800
C	-4.60668300	-0.75887700	0.44493900
C	-3.49883100	-1.45602300	0.95197000
C	-2.25317200	-0.83560300	1.03958200
C	1.25160300	-1.57819300	1.66415900
N	0.12991900	-1.01267800	1.20959500
C	-1.05524300	-1.51804700	1.59521900
C	-1.14680600	-2.60864800	2.47200200
C	0.02706100	-3.19763800	2.93499600
C	1.25365900	-2.67962900	2.52151600
C	-1.08605100	3.90373200	-1.00144100
C	-1.95416300	4.98724400	-1.09873100
C	-3.30088300	4.80196600	-0.77472100
C	-3.72950100	3.54390400	-0.36158700
C	-2.79941500	2.50013400	-0.30046500
N	-1.49791900	2.68232300	-0.62331700
C	-9.82912500	-3.30375900	-0.00820300
H	-7.05309200	0.36420500	0.84891100
H	-9.25726700	-0.70430900	0.62626100
H	-7.36255200	-4.40510300	-0.43907700
H	-5.15623200	-3.33395900	-0.25293900
H	-5.20608600	1.13793900	-0.39600300
H	-3.61830100	-2.48084400	1.28292800
H	2.17967600	-1.13341900	1.31778100
H	-2.11163300	-2.97412500	2.80567800
H	-0.01764700	-4.04243800	3.61589400
H	2.19212600	-3.11013100	2.85441600
H	-0.02364900	3.98872300	-1.21794000
H	-1.58007200	5.95404700	-1.41805200
H	-4.00210300	5.62915200	-0.82675400
H	-4.75954100	3.37896000	-0.06464200
Cu	-0.25461600	1.09755000	-0.09859200

C	5.83710900	-1.20550100	-0.24169900
C	6.96772500	-0.49897700	0.21702400
C	8.19362400	-1.12944500	0.41401500
C	8.36333700	-2.49383100	0.15117700
C	7.24787500	-3.20084700	-0.31825400
C	6.01770700	-2.57906100	-0.50515200
N	2.32883800	1.01890900	-0.98268100
C	2.98842500	1.23490200	0.27477400
C	4.17381300	0.54926800	0.43909000
C	4.54699000	-0.52067900	-0.42024600
C	3.52796200	-1.01949500	-1.26808400
C	2.34150200	-0.33741800	-1.45735300
C	-1.19223800	-0.63563700	-2.24286900
N	-0.04720400	-0.20419900	-1.68512900
C	1.11093500	-0.86936000	-1.98124200
C	1.07664000	-2.02147400	-2.80721800
C	-0.11822700	-2.46159000	-3.33758900
C	-1.29549100	-1.74546900	-3.06424500
C	0.30376500	2.93212800	2.03915400
C	0.91008100	3.55349100	3.11740000
C	2.29753100	3.39342800	3.26569600
C	2.99006700	2.63867900	2.34200800
C	2.30448400	2.02648800	1.26325000
N	0.95446000	2.18521700	1.12718100
C	9.68586100	-3.18323600	0.38663200
H	6.88786600	0.57052000	0.38908000
H	9.04208300	-0.54378200	0.76181500
H	7.34304900	-4.26351100	-0.53224700
H	5.17790500	-3.17917600	-0.84255600
H	4.74962200	0.70020800	1.34779400
H	3.62934400	-2.01485500	-1.68791600
H	-2.07625300	-0.05208600	-2.00248100
H	2.00191900	-2.53427200	-3.04342600
H	-0.14042700	-3.34036300	-3.97615300
H	-2.25381600	-2.03588300	-3.47981500
H	-0.76619000	3.02672700	1.88061700
H	0.32398700	4.14359900	3.81276800
H	2.82489300	3.86828700	4.08830100
H	4.06611600	2.53036000	2.41501800
H	-10.61058900	-2.58900500	-0.28249400
H	-10.11522100	-3.74686400	0.95382800
H	-9.82894200	-4.10862300	-0.74925700
H	9.83932700	-4.00856400	-0.31638700
H	9.74470100	-3.60575700	1.39844700

H	10.52507700	-2.48878100	0.27759100
C	2.10334700	2.09133500	-1.79983500
O	2.15456500	3.26697900	-1.45464600
O	1.73971900	1.72300300	-3.05932700
H	1.57270300	2.56073300	-3.51508900

$Cu(Tpy)_2$-CO

C	6.50805200	-0.55936300	0.08269100
C	7.19639000	-1.12677100	1.17254300
C	8.50625400	-1.58400200	1.03734600
C	9.18595200	-1.49957900	-0.18892000
C	8.49768400	-0.94070100	-1.27733100
C	7.18828600	-0.47852300	-1.14692200
C	2.88317800	-0.23107500	1.16301800
C	4.19225900	-0.71867000	1.07171600
C	5.11685500	-0.07242300	0.22629000
C	4.64910800	1.04317200	-0.48400200
C	3.32256100	1.47624800	-0.33380700
C	0.91256400	3.76507800	-1.82909200
N	1.45178500	2.76420800	-1.14394900
C	2.78891300	2.64424900	-1.09132400
C	3.62951400	3.57707200	-1.73223100
C	3.05844100	4.62693200	-2.44814700
C	1.66567300	4.72899700	-2.50870800
C	-0.34040000	-1.56037800	2.30900600
C	-0.05702000	-2.21304600	3.50885000
C	1.26000900	-2.20962200	3.97098800
C	2.23488200	-1.56268900	3.21355700
C	1.86939700	-0.92825400	2.01456300
N	0.59239600	-0.93145000	1.58324300
C	10.61364200	-1.96749700	-0.32021800
H	6.70703400	-1.18844100	2.14744100
H	9.01610600	-2.01236500	1.90536100
H	8.99317200	-0.87621400	-2.25037300
H	6.67404900	-0.07782300	-2.02349600
H	4.48241800	-1.62538700	1.60218100
H	5.32625500	1.57324500	-1.15350600
H	-0.18348700	3.80627600	-1.83495400
H	4.71493400	3.49655200	-1.66091700
H	3.69463600	5.36125900	-2.94889500
H	1.17457500	5.53366700	-3.06005000
H	-1.34934700	-1.53400800	1.89292700
H	-0.85736900	-2.70638700	4.06369400
H	1.52563600	-2.69825000	4.91189400

H	3.26958300	-1.52791400	3.55655300
Cu	-0.22309500	0.14788400	-0.10393700
C	-6.66852000	-0.29838600	-0.13538800
C	-7.43256100	0.88985100	-0.07985700
C	-8.82381100	0.86181600	-0.10297500
C	-9.53310300	-0.34964900	-0.18803500
C	-8.78093800	-1.53204300	-0.24623500
C	-7.38593800	-1.51075200	-0.22250700
N	-2.35328200	-0.26069300	-0.03047100
C	-3.07668500	0.75401600	0.55380500
C	-4.48041700	0.75222000	0.51994100
C	-5.19519100	-0.27478400	-0.10568000
C	-4.41384300	-1.30772800	-0.70777300
C	-3.02691500	-1.25834000	-0.64621400
C	-0.21279300	-3.51609700	-1.20770600
N	-0.99149800	-2.55708000	-0.70954800
C	-2.20277100	-2.34419300	-1.25234800
C	-2.65243300	-3.10320300	-2.35174700
C	-1.83310100	-4.10456900	-2.86824800
C	-0.58066100	-4.32692200	-2.28417600
C	-0.16497400	2.68128900	1.59091200
C	-0.65572600	3.73287200	2.35990600
C	-2.05260300	3.82012500	2.53568400
C	-2.87166100	2.86514300	1.95788500
C	-2.30364100	1.81567800	1.18975100
N	-0.94954200	1.76152900	1.02153100
C	-11.04087100	-0.36175400	-0.23345200
H	-6.92452800	1.85636200	-0.04670500
H	-9.37763800	1.80529400	-0.06635900
H	-9.29855700	-2.49463800	-0.30209900
H	-6.84446800	-2.45911900	-0.23904100
H	-5.02606900	1.55801500	1.00986400
H	-4.90636800	-2.13851900	-1.21042400
H	0.76500800	-3.64151700	-0.72584100
H	-3.61847500	-2.89017200	-2.81094900
H	-2.16165700	-4.69672000	-3.72639600
H	0.09684400	-5.09721100	-2.65912300
H	0.90256900	2.54653000	1.40267800
H	0.02640000	4.45787400	2.80735400
H	-2.48887500	4.62645800	3.13146400
H	-3.95055400	2.91393200	2.10794600
H	10.86394000	-2.22095100	-1.36168900
H	10.80893600	-2.85335000	0.30420400
H	11.31765000	-1.18068600	0.00394900

H	-11.44284200	-1.38114300	-0.12976500
H	-11.41683300	0.04720600	-1.18792700
H	-11.47365700	0.25609300	0.57106700
N	2.46503500	0.83866800	0.48144600
C	0.43349400	0.10860400	-1.83405300
O	0.79591100	0.08163400	-2.91622200

Cu(Tpy)$_2$-CO (Transition State)

C	6.02439900	-0.99516200	-0.49077200
C	6.84814600	-1.59799800	0.47739700
C	7.96341700	-2.34987500	0.10799100
C	8.30172000	-2.53872200	-1.24142900
C	7.48040000	-1.93573400	-2.20754000
C	6.36710500	-1.17885900	-1.84332900
C	2.90428300	0.22411100	1.31623400
C	4.04930900	-0.53425400	1.00377300
C	4.83775400	-0.19229800	-0.10167300
C	4.44109100	0.94064600	-0.83478200
C	3.28907400	1.64952900	-0.45675800
C	1.17831500	4.23583300	-1.95514900
N	1.57509300	3.15458500	-1.28694700
C	2.88383800	2.88065900	-1.20994700
C	3.84784900	3.71807900	-1.80305900
C	3.42710100	4.85172700	-2.49837100
C	2.06039500	5.12071100	-2.58477100
C	-0.00033800	-0.10442100	3.54331100
C	0.51740400	-0.59919400	4.73554900
C	1.88949700	-0.88394100	4.79562300
C	2.66891000	-0.63716800	3.67005100
C	2.07731000	-0.11720000	2.50328600
N	0.74295700	0.12273500	2.44479300
C	9.48811200	-3.38083000	-1.64081600
H	6.63062600	-1.45312600	1.53824200
H	8.59085400	-2.79313200	0.88676600
H	7.71360000	-2.06595700	-3.26839700
H	5.73834000	-0.74439400	-2.62396100
H	4.28847400	-1.42289100	1.58913400
H	5.02294200	1.26296000	-1.69821800
H	0.09647800	4.41069000	-1.99399500
H	4.91190800	3.49944800	-1.69995000
H	4.15942000	5.51912300	-2.96023700
H	1.68268200	5.99449700	-3.12049200
H	-1.06731400	0.11873200	3.44928700
H	-0.13830100	-0.75382600	5.59453100

H	2.34327800	-1.27266700	5.71034800
H	3.74620900	-0.80712500	3.70077600
Cu	-0.39507400	0.07461200	0.72449700
C	-6.51986400	-0.89293700	-0.65473400
C	-7.33798000	0.14057700	-1.15463000
C	-8.67428000	-0.08569700	-1.48897400
C	-9.26322900	-1.34966300	-1.34083000
C	-8.45203400	-2.38331900	-0.83946900
C	-7.11913900	-2.16299500	-0.50349400
N	-2.41918800	-0.21380400	0.39010900
C	-3.30298700	0.79638500	0.44903200
C	-4.64330500	0.62457400	0.12077400
C	-5.10312000	-0.65936100	-0.30176500
C	-4.14512500	-1.67713400	-0.40456500
C	-2.79845900	-1.43563900	-0.08114100
C	0.60207300	-2.74074200	-0.23280200
N	-0.43664200	-1.90611100	-0.08676800
C	-1.71201400	-2.39538000	-0.25110400
C	-1.91020300	-3.76117900	-0.56751000
C	-0.82519900	-4.60752500	-0.71686200
C	0.47721200	-4.08870900	-0.54660700
C	-0.73339200	3.26085700	1.25453200
C	-1.41767900	4.42717600	1.60112500
C	-2.81737900	4.40399300	1.57945700
C	-3.47088000	3.22911100	1.21238500
C	-2.70912900	2.09911300	0.86808400
N	-1.36046600	2.13686900	0.89844600
C	-10.70751700	-1.60111700	-1.69798500
H	-6.91551900	1.13478700	-1.31583200
H	-9.27194500	0.74144000	-1.88401800
H	-8.87939200	-3.38108000	-0.69810800
H	-6.53520200	-2.98717000	-0.08746300
H	-5.34323900	1.45606600	0.19175300
H	-4.44105000	-2.65135600	-0.79497400
H	1.59457200	-2.29614800	-0.09978000
H	-2.92324700	-4.14956000	-0.68355800
H	-0.97814100	-5.66211700	-0.95970900
H	1.36427800	-4.71478400	-0.65821000
H	0.35995200	3.21138900	1.24638800
H	-0.86720800	5.32765500	1.88241600
H	-3.39415500	5.29174200	1.85199100
H	-4.56070100	3.18448700	1.20553800
H	10.26041800	-3.39029300	-0.85669600
H	9.94908800	-3.01576200	-2.57154000

H	9.18862500	-4.42929900	-1.81649300
H	-10.80191300	-2.38043800	-2.47349700
H	-11.19457200	-0.69070300	-2.07812000
H	-11.28451700	-1.94976800	-0.82426600
N	2.52620800	1.28174600	0.57872400
C	0.05168400	0.48273600	-2.07142800
O	0.99215500	0.05876600	-2.54455200

References

1. Zhang, X., Wang, Y., Gu, M., Wang, M., Zhang, Z., Pan, W., Jiang, Z., Zheng, H., Lucero, M., Wang, H., Sterbinsky, G. E., Ma, Q., Wang, Y.-G., Feng, Z., Li, J., Dai, H. & Liang, Y. Molecular engineering of dispersed nickel phthalocyanines on carbon nanotubes for selective CO_2 reduction. *Nat. Energy* **5**, 684-692 (2020).
2. Zhang, X., Wu, Z., Zhang, X., Li, L., Li, Y., Xu, H., Li, X., Yu, X., Zhang, Z., Liang, Y. & Wang, H. Highly selective and active CO_2 reduction electrocatalysts based on cobalt phthalocyanine/carbon nanotube hybrid structures. *Nat. Commun.* **8**, 14675 (2017).
3. Zhu, H., Lu, M., Wang, Y., Yao, S., Zhang, M., Kan, Y., Liu, J., Chen, Y., Li, S. & Lan, Y. Efficient electron transmission in covalent organic framework nanosheets for highly active electrocatalytic carbon dioxide reduction. *Nat. Commun.* **11**, 497-10 (2020).
4. Xin, Z., Wang, Y., Chen, Y., Li, W., Dong, L. & Lan, Y. Metallocene implanted metalloporphyrin organic framework for highly selective CO_2 electroreduction. *Nano Energy* **67**, 104233 (2020).
5. Han, N., Wang, Y., Ma, L., Wen, J., Li, J., Zheng, H., Nie, K., Wang, X., Zhao, F., Li, Y., Fan, J., Zhong, J., Wu, T., Miller, D., Lu, J., Lee, S. & Li, Y. Supported cobalt polyphthalocyanine for high-performance electrocatalytic CO_2 reduction. *Chem* **3**, 652-664 (2017).
6. Sun, L., Huang, Z., Reddu, V., Su, T., Fisher, A. & Wang, X. A planar, conjugated N_4-macrocyclic cobalt complex for heterogeneous electrocatalytic CO_2 reduction with high activity. *Angew. Chem. Int. Ed*. **59**, 17104-17109 (2020).
7. Wang, J., Huang, X., Xi, S., Xu, H. & Wang, X. Axial modification of cobalt complexes on heterogeneous surface with enhanced electron transfer for carbon dioxide reduction. *Angew. Chem. Int. Ed*. **59**, 19162-19167 (2020).
8. Wang, M., Torbensen, K., Salvatore, D., Ren, S., Joulié, D., Dumoulin, F., Mendoza, D., Lassalle-Kaiser, B., Işci, U., Berlinguette, C. & Robert, M. CO_2 electrochemical catalytic reduction with a highly active cobalt phthalocyanine. *Nat. Commun.***10**, 3602-3608 (2019).
9. Zhu, M., Ye, R., Jin, K., Lazouski, N. & Manthiram, K. Elucidating the reactivity and mechanism of CO_2 electroreduction at highly dispersed cobalt phthalocyanine. *ACS Energy Lett.* **3**, 1381-1386 (2018).
10. Choi, J., Wagner, P., Gambhir, S., Jalili, R., MacFarlane, D., Wallace, G. & Officer, D. Steric modification of a cobalt phthalocyanine/graphene catalyst to give enhanced and stable electrochemical CO_2 reduction to CO. *ACS Energy Lett.* **4**, 666-672 (2019).
